# The ErbB2–Dock7 Signaling Axis Mediates Excessive Cell Morphogenesis Induced by Autism Spectrum Disorder- and Intellectual Disability-Associated Sema5A p.Arg676Cys

**DOI:** 10.3390/ijms262110656

**Published:** 2025-11-01

**Authors:** Mikito Takahashi, Hideji Yako, Ayaka Suzuki, Ryuma Isa, Yuki Miyamoto, Junji Yamauchi

**Affiliations:** 1 Laboratory of Molecular Neuroscience and Neurology, Tokyo University of Pharmacy and Life Sciences, Hachioji, Tokyo 192-0392, Japanhyako@toyaku.ac.jp (H.Y.); miyamoto-y@ncchd.go.jp (Y.M.); 2 Department of Pharmacology, National Research Institute for Child Health and Development, Setagaya, Tokyo 157-8535, Japan; 3 Department of Biological Sciences, Tokyo College of Biotechnology, Ota, Tokyo 157-8535, Japan

**Keywords:** Sema5A, ASD, ID, ErbB2, Dock7, morphogenesis

## Abstract

Characterized by social communication deficits and the presence of restricted and repetitive behaviors, autism spectrum disorder (ASD) is a significant neurodevelopmental condition. Genetic studies have revealed a strong association between ASD and numerous mutations that alter the function of key proteins, either through activation or inactivation. These alterations are widely hypothesized to affect neuronal morphogenesis; however, a comprehensive understanding of the specific molecular cascades driving these cellular and symptomatic changes remains lacking. In this study, we report for the first time that signaling through the atypical Rho family guanine-nucleotide exchange factor (GEF) Dock7 and ErbB2, an activator acting upstream of Dock7, drives the excessive elongation of neuronal processes observed in association with the ASD- and intellectual disability (ID)-linked semaphorin-5A (Sema5A) Arg676Cys variant (p.Arg676Cys). Knockdown of Dock7 using short hairpin RNA or inhibition of ErbB2 kinase signaling with a specific chemical inhibitor reduced this excessive process elongation in primary cortical neurons. Similar results were obtained in the N1E-115 cell line, a neuronal cell model that undergoes neuronal morphological differentiation. Moreover, inhibition of ErbB2-Dock7 signaling specifically decreased the overactivation of the downstream molecules Rac1 and Cdc42. These findings indicate that the ErbB2–Dock7 signaling axis plays a role in mediating the aberrant neuronal morphology associated with the ASD- and ID-linked Sema5A p.Arg676Cys. Targeting this pathway may therefore offer a potential approach to addressing the molecular and cellular developmental challenges observed in ASD.

## 1. Introduction

Autism is classified as a developmental disability and is now more commonly referred to as autism spectrum disorder (ASD). ASD encompasses autism, pervasive developmental disorder, and Asperger syndrome. The primary characteristics of ASD include difficulties in social interaction and communication, along with repetitive behaviors and a strong attachment to specific objects [1,2,3,4]. Individuals with ASD often exhibit hypersensitivity to sensory stimuli such as light and sound, although some may display hyposensitivity instead. Accordingly, ASD can be described as a neuro-integrative disorder affecting the sensory system, involving the central nervous system (CNS) and, in some cases, the peripheral nervous system (PNS) [1,2,3,4].

The causes of ASD are complex, involving multiple factors, including both genetic and environmental components. A large proportion of ASD cases, estimated at 50% to 90%, are associated with either inherited or spontaneous gene mutations. These mutations frequently result in amino acid changes that can alter catalytic activities or binding properties, leading to either a gain or loss of function [5,6,7,8]. Additionally, such alterations may influence post-translational modifications, protein stability, and the localization of proteins within the cell. These genetic changes are believed to disrupt neuronal development, particularly during neurite formation, and may also affect earlier phases of neurogenesis [6,7].

During CNS development, neuronal cells undergo continuous and dynamic morphological changes [9,10]. These processes include neurite initiation and elongation, directional guidance of neuronal processes, and the establishment of neuronal networks through synaptogenesis [11,12]. Despite extensive research, the molecular mechanisms regulating the distinct stages of neuronal morphological differentiation remain incompletely understood [11,12,13,14]. In neurological disorders, neuronal morphogenesis can be impaired not only during early development but also at later, and even very early, stages of life [7,8,9,10].

The transmembrane protein semaphorin-5A (Sema5A) functions as an axon guidance cue, controlling growth cone extension, neuronal process elongation, and directional navigation [15,16,17,18]. Sema5A exerts dual functions, acting either as a ligand or receptor depending on the developmental stage and cell type [19,20,21,22]. Its expression, both in terms of presence and abundance, can vary according to cell type and developmental timing [19,20,21,22]. Importantly, mutations in the gene encoding Sema5A have been strongly associated with autism spectrum disorder (ASD) and, in some cases, with intellectual disability (ID), which is characterized by below-average cognitive performance along with deficits in conceptual and social communication [23,24,25,26]. Such mutations have also been linked to epilepsy [27,28]. These neurological conditions are thought to result from abnormal neuronal connectivity, caused either by impaired synapse formation or by defective elongation of neuronal processes required to establish functional circuits in specific brain regions [29,30].

We previously reported that the ASD- and ID-associated Sema5A Arg676-to-Cys variant (p.Arg676Cys) induces excessive neuronal processes [25,31,32] through a signalosome composed of genetically conserved molecules, including the adaptor protein Elmo2 (also known as the Ced-12 orthologue) and Dock5 (also known as the Ced-5 orthologue), a Dock-family guanine-nucleotide exchange factor (GEF) specific for the Rho family small GTPase Rac1 [33,34,35,36]. In this study, we show that Sema5A p.Arg676Cys also mediates a genetically distinct signaling pathway through ErbB2 and Dock7, resulting in excessive process elongation through Rac1 and/or Cdc42 [37,38] in both cortical neurons [39,40] and N1E-115 cells, a neuronal model that undergoes morphological differentiation [41,42]. Dock7, a member of the Dock-C family GEFs, is dually specific for the Rho GTPases Rac1 and/or Cdc42 [34,36,37,38] and acts downstream of ErbB2 kinase [34,38]. Knockdown of Dock7 using short hairpin RNA (shRNA) or inhibition of ErbB2 kinase activity restored normal cell morphology as well as Rac1 and Cdc42 activities. These findings identify Dock7 as a key mediator of Sema5A p.Arg676Cys-induced neuronal cell morphogenesis, highlighting a previously unrecognized specific signaling axis in this disorder at both the molecular and cellular levels.

## 2. Results

### 2.1. Knockdown of Dock7 Protein Decreases Mutated Sema5A-Induced Phenotypes

First, to investigate whether Dock7 [33,34,35,36], a Dock-C family member molecule distinct from Dock-A family members, contributes to the phenotypes of excessive neuronal process elongation induced by the ASD- and ID-associated Sema5A p.Arg676Cys variant [25,31,32], we focused on Dock7 because of its established role in axonal formation [34,37]. We confirmed that excessive process elongation is specifically induced by Sema5A p.Arg676Cys [25,31,32] (Figure S1). Under these conditions, the expression levels of wild type Sema5A and Sema5A p.Arg676Cys were comparable (Figure S2). Following transfection with Sema5A p.Arg676Cys, Tyr-1118 of Dock7 was phosphorylated, whereas transfection with wild type Sema5A did not induce this phosphorylation (Figure S3). Phosphorylation of Tyr-1118 of Dock7 is known to activate Dock7 [38].

**Figure 1:**
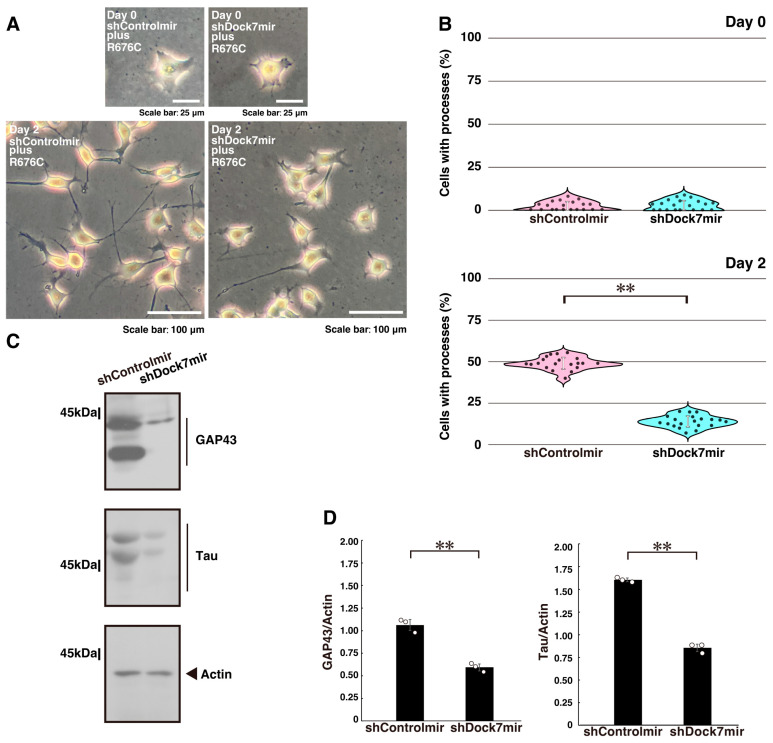
Knockdown of Dock7 recovers mutated Sema5A-induced excessive process elongation. (**A**,**B**) N1E-115 cells harboring Sema5A p.Arg676Cys (R676C) were transfected with plasmids encoding shRNA targeting Dock7 (shDock7mir) or control luciferase (shLuciferase[Control]mir) and cultured for 0 or 2 days following the induction of differentiation. Representative images are shown. Cells with processes with a body length of more than one cell were counted as exhibiting neurite-like process elongation and are statistically depicted in the graph (** *p* < 0.01; *n* = 20 fields). (**C**,**D**) At day 3 following the induction of differentiation, cell lysates from cells were immunoblotted using antibodies against neuronal differentiation marker proteins GAP43 and Tau or the internal control marker protein actin. Immunoreactive band values are calculated by dividing the band intensities of the marker protein by those of the actin protein (** *p* < 0.01; *n* = 3 blots).

To further test Dock7’s role, we transfected N1E-115 cells, a model for neuronal differentiation, with a plasmid encoding Dock7 shRNA to knock down Dock7 (Figure S4). Knockdown of Dock7 significantly reduced the excessive process elongation (Figure 1A,B). Additionally, the expression levels of neuronal differentiation markers, growth-associated protein 43 (GAP43) and Tau, were downregulated following Dock7 knockdown, while the expression of the control protein actin remained comparable (Figure 1C,D).

We then performed the same knockdown experiments in primary cortical neurons expressing Sema5A p.Arg676Cys, which exhibit elongated processes. As expected, Dock7 knockdown similarly reduced process elongation (Figure S5). Collectively, these results indicate that Dock7 contributes to the excessive elongation of neuronal processes.

### 2.2. Inhibition of ErbB2 or Its Interaction with Dock7 Decreases Mutated Sema5A-Induced Phenotypes

**Figure 2:**
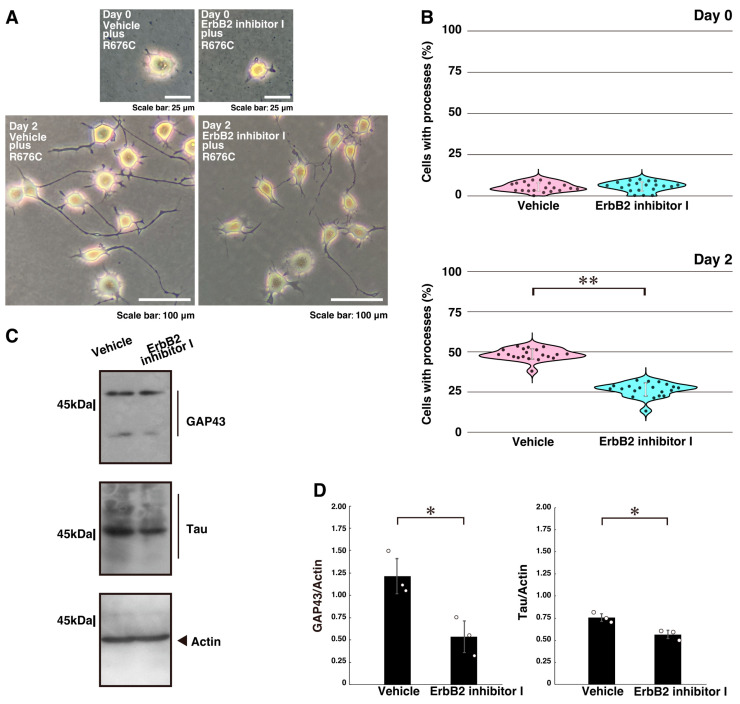
Treatment with an ErbB2 inhibitor I recovers mutated Sema5A-induced excessive process elongation. (**A**,**B**) N1E-115 cells harboring Sema5A p.Arg676Cys (R676C) were treated with the ErbB2 inhibitor I (also called inhibitor I or AG825, 0.01 mM) or its vehicle and cultured for 0 or 2 days following the induction of differentiation. Representative images are shown. Cells with processes with a body length of more than one cell were counted as exhibiting neurite-like process elongation and are statistically depicted in the graph (** *p* < 0.01; *n* = 20 fields). (**C**,**D**) At day 3 following the induction of differentiation, cell lysates were immunoblotted using antibodies against neuronal differentiation marker proteins GAP43 and Tau or the internal control marker protein actin. Immunoreactive band values are calculated by dividing the band intensities of the marker protein by those of the actin protein (* *p* < 0.05; *n* = 3 blots).

Next, we examined whether ErbB2 kinase, an upstream regulator of Dock7 [38], participates in the excessive process elongation induced by Sema5A p.Arg676Cys. N1E-115 cells were treated with the chemical ErbB2 inhibitor AG825. Treatment with the ErbB2 inhibitor, but not with vehicle controls, reversed the excessive neurite elongation (Figure 2A,B). Similar effects were observed in primary cortical neurons (Figure S5). These changes were accompanied by decreased expression levels of the neuronal differentiation markers GAP43 and Tau following ErbB2 inhibitor treatment (Figure 2C,D).

**Figure 3:**
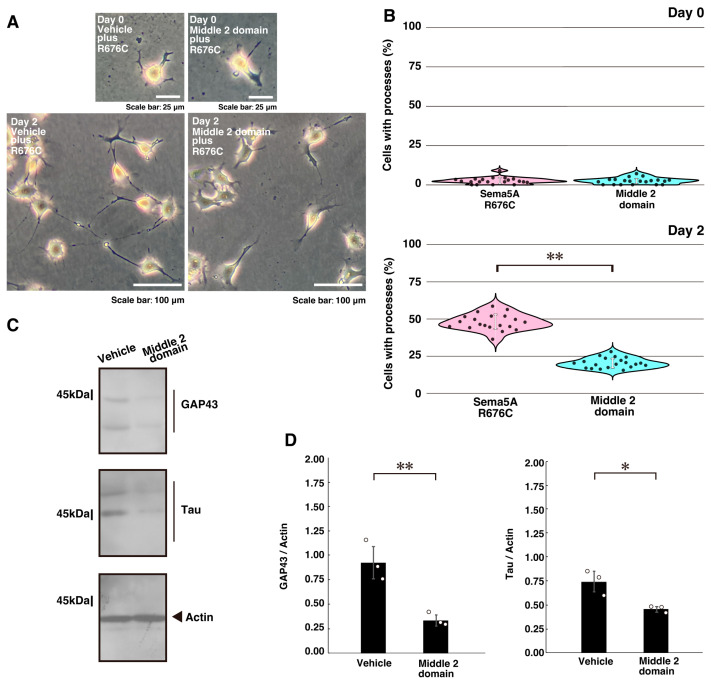
Transfection of ErbB2 interactive domain recovers mutated Sema5A-induced excessive process elongation. (**A**,**B**) N1E-115 cells harboring Sema5A p.Arg676Cys (R676C) were transfected with the plasmid encoding the ErbB2 interactive domain (Dock7’s middle 2 domain [amino acids 1111 to 1431 of Dock7]) or a control (mock) plasmid and cultured for 0 or 2 days following the induction of differentiation. Representative images are shown. Cells with processes with a body length of more than one cell were counted as exhibiting neurite-like process elongation and are statistically depicted in the graph (** *p* < 0.01; *n* = 20 fields). (**C**,**D**) At day 3 following the induction of differentiation, the lysates from cells were immunoblotted using antibodies against neuronal differentiation marker proteins GAP43 and Tau or the internal control marker protein actin. Immunoreactive band values are calculated by dividing the band intensities of the marker protein by those of the actin protein (** *p* < 0.01 and * *p* < 0.05; *n* = 3 blots).

We then transfected N1E-115 cells with a plasmid encoding the Dock7 region that interacts with ErbB2 (middle 2 domain; amino acids 1111 to 1431 of Dock7) [34,38]. Transfection with this interactive region, but not with control plasmids, reversed the excessive process elongation (Figure 3A,B). Similar results were observed in cortical neurons (Figure S5), accompanied by decreased expression of GAP43 and Tau (Figure 3C,D). Together, these findings indicate that the interaction between ErbB2 and Dock7 contributes to the excessive elongation of neuronal processes.

### 2.3. Downstream Effectors Rac1 and Cdc42 Contribute to Mutated Sema5A-Induced Phenotypes

**Figure 4:**
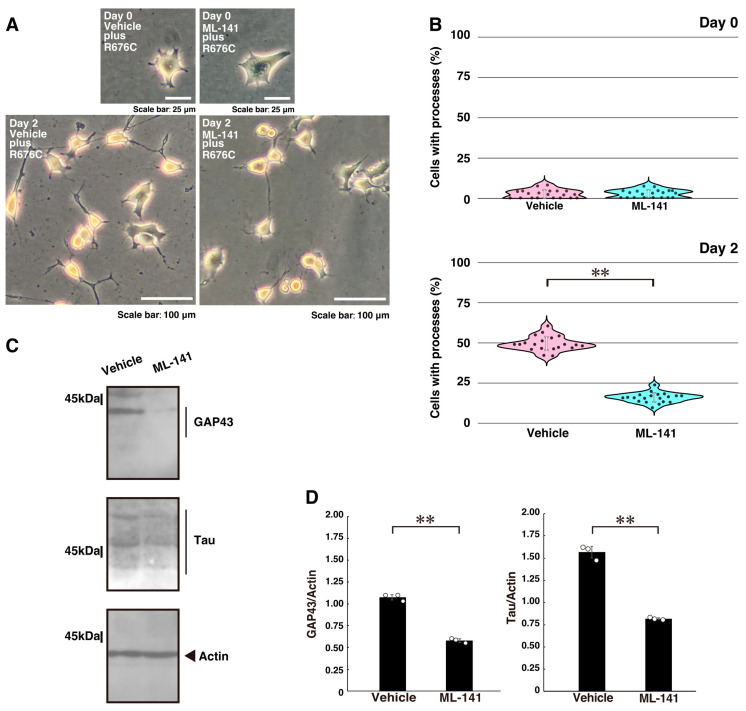
Treatment with a Rac1 and Cdc42 inhibitor recovers mutated Sema5A-induced excessive process elongation. (**A**,**B**) N1E-115 cells harboring Sema5A p.Arg676Cys (R676C) were treated with the Rac1 and Cdc42 inhibitor (ML-141, 0.01 mM) or its vehicle and cultured for 0 or 2 days following the induction of differentiation. Representative images are shown. Cells with processes with a body length of more than one cell were counted as exhibiting neurite-like process elongation and are statistically depicted in the graph (** *p* < 0.01; *n* = 20 fields). (**C**,**D**) At day 3 following the induction of differentiation, the lysates from cells were immunoblotted using antibodies against neuronal differentiation marker proteins GAP43 and Tau or the internal control marker protein actin. Immunoreactive band values are calculated by dividing the band intensities of the marker protein by those of the actin protein (** *p* < 0.01; *n* = 3 blots).

Finally, we investigated whether the downstream Dock7 molecules Rac1 and Cdc42 [33,34,35,36] contribute to the excessive process elongation induced by Sema5A p.Arg676Cys. Treatment of N1E-115 cells with the chemical Rac1 and Cdc42 inhibitor ML-141 reduced excessive process elongation (Figure 4A,B) and decreased the expression levels of GAP43 and Tau (Figure 4C,D), consistent with the reduction in process elongation observed in cortical neurons (Figure S5).

**Figure 5:**
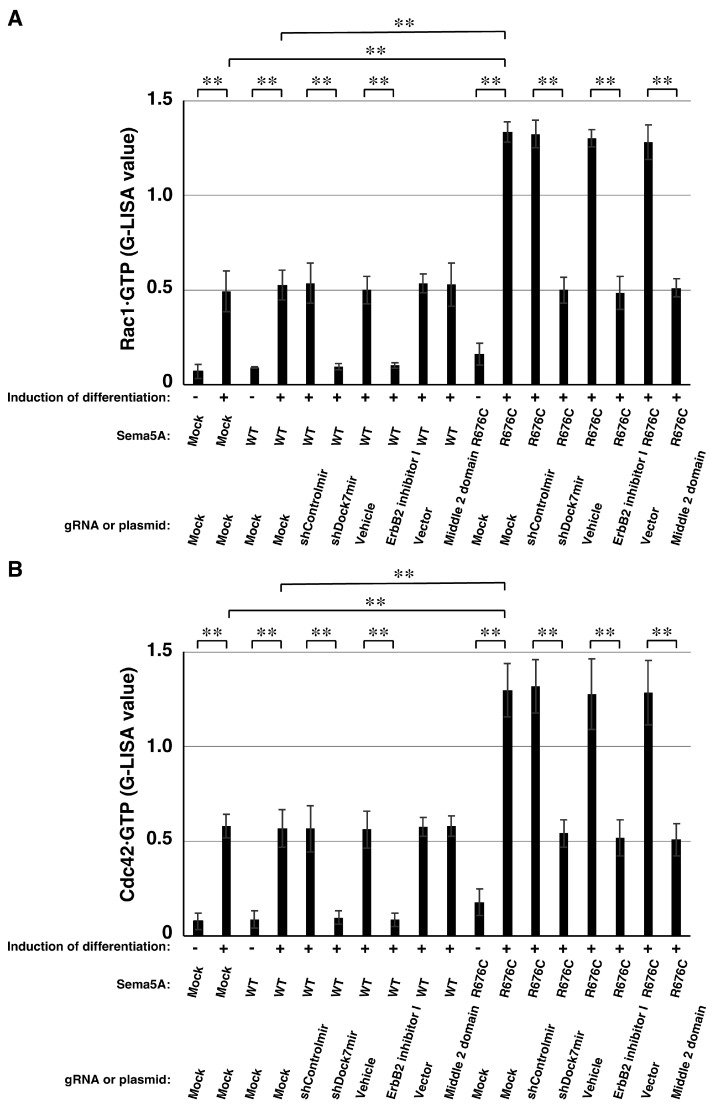
Sema5A p.Arg676Cys specifically activates Rac1 and Cdc42 through ErbB2 and Dock7. (**A**,**B**) Parental cells transfected with mock plasmid or cells harboring wild type Sema5A (WT) or Sema5A p.Arg676Cys (R676C) were transfected with plasmids encoding the respective plasmids or were treated with the respective chemical inhibitors and cultured for 2 days with (+) or without (−) differentiation induction. Cell lysates were analyzed using a G-LISA GTPase kit to assay the amounts of GTP-bound Rac1 or Cdc42 (** *p* < 0.01; *n* = 3).

We then assessed whether Rac1 and Cdc42 are activated in the presence of wild type Sema5A or Sema5A p.Arg676Cys in N1E-115 cells. Increased levels of GTP-bound Rac1 and Cdc42 in cells expressing Sema5A p.Arg676Cys were decreased by Dock7 knockdown, interactive domain transfection, or inhibition of ErbB2 or Rac1/Cdc42 (Figure 5A,B). In contrast, the levels of GTP-bound forms of Rac1 and Cdc42 in cells expressing wild type Sema5A were only modestly decreased by Dock7 knockdown or inhibition of ErbB2. These results indicate that, in the pathway downstream of Sema5A p.Arg676Cys, Rac1 and Cdc42 specifically function downstream of the ErbB2–Dock7 signaling axis

## 3. Discussion

In this study, we identify a previously unrecognized signaling axis involving ErbB2 and Dock7 that mediates the excessive neuronal process elongation induced by the ASD- and ID-associated Sema5A p.Arg676Cys variant (Figure S6) [25,31,32]. Our results provide mechanistic insight into how a single amino acid substitution in Sema5A can profoundly alter neuronal cell morphological changes. These findings also extend earlier studies implicating a signalosome containing Elmo2 and Dock5 in the pathological signaling induced by Sema5A p.Arg676Cys [31,32]. We hypothesize that Sema5A p.Arg676Cys engages two distinct Dock family GEF pathways: the Elmo2-Dock5 route (a genetically conserved Dock-A family signaling) and the ErbB2-Dock7 pathway (a genetically divergent Dock-C family signaling) [33,34,35,36]. This conclusion is supported by the knockdown or inhibition of these molecules in both neuronal cell lines and primary cultures.

Our findings highlight the complexity of disease-related Sema5A signaling in neuronal morphological changes and suggest that the activities of Rac1 and/or Cdc42, in addition to the established roles of Rho GTPase RhoA in neuronal pathological signaling, may be critical for the abnormal cellular morphologies observed [43,44,45,46]. Although our study demonstrates phenotypic effects following knockdown of the target genes, we did not perform rescue experiments to confirm restoration of mRNA expression levels. Such experiments would be valuable to further assess the specificity of the observed phenotypes and to exclude possible off-target effects of shRNA. Future studies could investigate whether gene reintroduction restores normal neuronal morphology.

The role of Sema5A in neuronal process guidance and network formation is well documented [29,30]; however, certain mutations in the gene encoding Sema5A are increasingly recognized as risk or casual factors for ASD and ID [21,22,23,24]. These clinical associations suggest that disrupted neuronal process morphogenesis or connectivity is a core pathogenic feature [29,30], but the detailed molecular connections linking Sema5A p.Arg676Cys to abnormal cell morphogenesis have remained unclear. Our results address this gap by providing evidence that ErbB2-Dock7 signaling, in addition to Elmo2-Dock5 signaling, drives excessive elongation of neuronal processes. Notably, the morphological changes observed in the presence of Sema5A p.Arg676Cys, accompanied by upregulation of differentiation markers, are reversed by Dock7 knockdown or ErbB2 inhibition, thereby establishing a causal role for this signaling axis.

The involvement of Dock7 in this context is particularly significant. Dock7, a member of the genetically divergent Dock-C family [33,34,35,36], is known to regulate neuronal polarization and specification mainly through activation of Rac1 [37]. In this process, Dock7 coordinates actin and tubulin cytoskeletal remodeling, controlling neurite polarization and specification [37]. By positioning Dock7 downstream of ErbB2 in the pathway triggered by Sema5A p.Arg676Cys, our study reveals a novel regulatory layer linking the extracellular receptor tyrosine kinase to intracellular cytoskeletal machinery. Given that aberrant cytoskeletal remodeling is implicated in multiple neurodevelopmental disorders [43,44,45,46], these findings suggest that the ErbB2–Dock7 signaling axis may represent a common pathway through which distinct genetic alterations converge to produce similar cellular outcomes.

It is also important to consider the upstream role of ErbB2. Although traditionally studied in the context of cell proliferation and tumorigenesis [47,48], emerging evidence indicates that ErbB2 exerts diverse functions in the nervous system, including promoting neuronal cell survival, synaptic plasticity, and glial regulation [49,50]. Dysregulation of ErbB2 signaling has been implicated in neurodevelopmental disorders [51,52], raising the possibility that ErbB2 may contribute more broadly to abnormalities in neural morphogenesis beyond the context of Sema5A mutations.

Taken together, these findings suggest that Rho family small GTPases downstream of Sema5A p.Arg676Cys may act as a pathological hub by engaging both classical Elmo2-Dock5 signaling and nonclassical ErbB2-Dock7 signaling. These alterations could disrupt proper neuronal circuit formation during brain development, thereby contributing to the features of ASD and ID. This framework may provide a mechanistic link between subtle genetic variations in Sema5A and broader connectivity deficits in the CNS.

The potential translational relevance of our results lies in the modulation of Dock7 in diseases associated with Sema5A mutations. Although Dock7 has been less extensively characterized than classical kinases, accumulating evidence supports its role in regulating neuronal cytoskeletal dynamics [33,34,37]. It should be noted, however, that broad inhibition of Dock7 may carry risks due to its involvement in multiple cellular functions and widespread tissue expression, including in the brain (see the Human Protein Atlas website, https://www.proteinatlas.org/ accessed on 30 October 2025). Nevertheless, our observations raise the possibility that future inhibitors or tailored compounds could be developed to selectively modulate specific pathological cellular signaling pathways.

A second potential implication of our results is the possibility of targeting ErbB2 in diseases involving Sema5A mutations. Inhibitors of ErbB2 kinase have been extensively studied and applied in contexts such as modulating abnormal cell proliferation during tumorigenesis [47,48], with their effects and mechanisms relatively well characterized. While broad inhibition of ErbB2 carries inherent risks due to its diverse and essential physiological roles, our findings suggest that carefully modified inhibitors, derivatives, or tailored compounds could potentially be explored in the future to selectively influence neuronal signaling pathways. Although such applications remain highly exploratory, the current observations provide a valuable conceptual framework to guide future research into the broader biological and translational implications of ErbB2 modulation in the nervous system.

In this context, specificity and potential side effects of targeting ErbB2 in the CNS must be considered. While ErbB2 inhibitors such as lapatinib and neratinib are approved and well characterized for cancer therapy, their blood–brain barrier permeability and neuronal specificity remain limited [47,48], raising concerns about off-target effects on glial and neuronal viability. Recent efforts to develop brain-penetrant ErbB2 inhibitors or targeted delivery systems, however, may provide a promising framework for future translational approaches [53,54]. Similarly, modulation of Dock7 activity would require highly selective compounds or allosteric modulators to avoid disrupting its essential roles in neuronal differentiation and other cellular processes. Therapeutic strategies targeting the ErbB2–Dock7 signaling axis will therefore need to balance efficacy with selectivity and safety, particularly in the CNS.

In conclusion, the present study identifies a specific role for the ErbB2–Dock7 signaling axis in mediating the excessive neuronal process elongation associated with the ASD- and ID-linked Sema5A p.Arg676Cys variant. These observations provide preliminary mechanistic insight into how a single amino acid substitution in Sema5A can influence neuronal morphology, complementing the previously described Elmo2-Dock5 pathway. We note that our experiments were conducted exclusively in the N1E-115 cell line; however, evidence from other neuronal cell lines and primary neuronal cultures supports similar or complementary observations [55,56,57]. Including such comparisons in future studies will help confirm and broaden our understanding of these mechanisms.

Additional studies are needed to elucidate not only the detailed molecular mechanisms by which Sema5A p.Arg676Cys links ErbB2 and Dock7, but also whether this pathway operates in human disease models. Further investigations are planned to evaluate the in vivo relevance of the ErbB2–Dock7 signaling axis. In particular, we are generating mouse models expressing the Sema5A p.Arg676Cys variant to determine whether this signaling pathway contributes to neuronal morphological abnormalities, defective process elongation, and connectivity defects in vivo. Such studies will be critical to assess whether the molecular mechanisms identified in our in vitro systems operate in the developing brain. While therapeutic applications targeting these molecules in diseases involving Sema5A mutations remain speculative at this stage, our findings provide a conceptual framework that may guide future research into the cell biological and translational implications of ErbB2 and/or Dock7 modulation within the CNS.

## 4. Materials and Methods

### 4.1. Key Antibodies and Plasmids

Key antibodies and plasmids used in this study are listed in Table 1.

### 4.2. Primary Cell Cultures

Animals were handled in accordance with the ARRIVE guidelines (https://arriveguidelines.org/ accessed on 30 October 2025), and all studies were conducted under a protocol reviewed and approved by the Tokyo University of Pharmacy and Life Sciences Animal Care and Use Committee (Approval Nos. L25-09 and L25-09). Mice were euthanized by chemical anesthesia via intraperitoneal injection of a mixture of 0.3 mg/kg medetomidine, 4 mg/kg midazolam, and 5 mg/kg butorphanol.

Primary cortical neuronal cells were isolated from the cerebrum of C57BL/6JJcl mice (Clea Japan, Inc., Tokyo, Japan) at embryonic days 16 to 17 and cultured as previously described [39,40]. Cells were maintained in culture without cryopreservation in Neurobasal medium (Gibco brand, Thermo Fisher Scientific, Waltham, MA, USA) supplemented with 2% B27 (Gibco brand, Thermo Fisher Scientific), 1% GlutaMAX (Gibco brand, Thermo Fisher Scientific), and 0.1 mg/mL gentamicin solution (Gibco brand, Thermo Fisher Scientific). Cells were maintained in 5% CO_2_ at 37 °C.

To observe process elongation, cells were allowed to extend processes for several days. On day 3 after seeding, cells with processes longer than two cell body lengths were considered process-bearing [39,40]. Process lengths were measured using Fiji software (ver. MacOS X86-64, https://imagej.net/software/fiji/ accessed on 30 October 2025). Under these conditions, fewer than 5% of attached cells incorporated trypan blue in each experiment.

### 4.3. Cell Line Cultures

The mouse neuronal N1E-115 cell line (JCRB, Osaka, Japan) was cultured on cell culture dishes (Nunc brand, Thermo Fisher Scientific) in high-glucose Dulbecco’s modified Eagle medium (DMEM; Nacalai Tesque, Kyoto, Japan; Fujifilm Wako Chemicals, Tokyo, Japan) containing 10% heat-inactivated fetal bovine serum (FBS) (Gibco brand, Thermo Fisher Scientific) and penicillin-streptomycin solution (Nacalai Tesque) in 5% CO_2_ at 37 °C [41,42].

Cells stably expressing either wild type *Sema5a* gene (WT) or the p.Arg676Cys mutant (R676C) were selected as clones in the presence of the antibiotic G418 (0.5 mg/mL, Nacalai Tesque) for 2 weeks [31,32], in accordance with the manufacturer’s instructions. These cells were maintained in culture without cryopreservation.

To induce differentiation, cells were cultured in DMEM and 1% FBS containing penicillin-streptomycin solution in 5% CO_2_ at 37 °C for several days, unless otherwise indicated. Cells with processes longer than one cell body were considered the longest process-bearing, differentiated cells (i.e., differentiated cells) [41,42]. The length of the longest process following the induction of differentiation was measured using Fiji software. Under these conditions, fewer than 5% of attached cells incorporated trypan blue (Nacalai Tesque) in each experiment.

### 4.4. Transfection of Plasmids

Cells were transfected with plasmids using ScreenFect A (Fujifilm) in accordance with the manufacturer’s instructions. The plasmid encoded either shRNA targeting luciferase (control) or Dock7 for, or the ErbB2-interactive domain of Dock7 (middle 2 domain; amino acids 1111–1431). The culture medium was replaced 4 h after transfection, and cells were typically used for 48 h or longer for cell biological and biochemical experiments, unless otherwise specified. Under these conditions, fewer than 5% of attached cells incorporated trypan blue in each experiment.

### 4.5. Microscopic Images

Multiple sets of transfected cells were microscopically observed using the i-NTER system (Micronet, Inc., Tokyo, Japan) and analyzed with i-NTER SHOT 2 software (ver. 2025, Micronet, Inc.). I In some experiments, cells on coverslips were fixed with 4% paraformaldehyde (Nacalai Tesque) or 100% cold methanol (Nacalai Tesque) and blocked with Blocking One (Nacalai Tesque). Slides were incubated with primary specific antibodies preloaded with fluorescent dye-conjugated secondary antibodies. Coverslips were mounted using Vectashield (Vector Laboratories, Burlingame, CA, USA). Fluorescent images were collected and merged using an FV3000 or FV4000 microscopic system equipped with a laser scanning Fluoview apparatus (Olympus, Tokyo, Japan) or a BZ-X700 microscopic system equipped with a fluorescence apparatus (Keyence, Tokyo, Japan). The images shown in the figures are representative of multiple observations and were analyzed using Fiji software.

### 4.6. Polyacrylamide Gel Electrophoresis and Immunoblotting

Cells were lysed in lysis buffer (50 mM HEPES-NaOH, pH 7.5, 150 mM NaCl, 5 mM MgCl_2_, 1 mM dithiothreitol, 1 mM phenylmethane sulfonylfluoride, 0.02 mM leupeptin, 1 mM EDTA, 1 mM Na_3_VO_4_, 10 mM NaF, and 0.5% NP-40) [31,32]. For standard denaturing conditions, centrifugally collected cell supernatants were denatured in sample buffer (Fujifilm Wako Chemicals). The samples were separated on a sodium dodecylsulfate polyacrylamide (SDS-PAGE) gel (Nacalai Tesque). Electrophoretically separated proteins were transferred to a polyvinylidene fluoride (PVDF) membrane (Merck & Co., Inc., Rahway, NJ, USA), blocked with Blocking One (Nacalai Tesque), and immunoblotted using primary antibodies, followed by peroxidase enzyme-conjugated secondary antibodies. Peroxidase-reactive bands were detected using X-ray film (Fujifilm) or TMB solution (Nacalai Tesque) [31,32], captured using a CanoScan LiDE4000 (Canon, Tokyo, Japan), and analyzed with CanoScan software (ver. 2024, Canon). The blots shown in the figures are representative of three independent experiments. Immunoreactive bands were quantified relative to control bands using Fiji software.

### 4.7. Assay for GTP-Bound Rac1 or Cdc42

Cells were lysed in G-LISA cell lysis buffer according to the manufacturer’s instructions (G-LISA GTPase kit, Cytoskeleton, Inc., Denver, CO, USA). Briefly, centrifugally collected cell supernatants (equal amounts of samples) were incubated in G-LISA affinity capture wells. After washing, wells were incubated with the respective specific antibodies. Following additional washes, wells were incubated with peroxidase enzyme-conjugated secondary antibodies and colorimetrically detected using peroxidase reaction reagents.

### 4.8. Statistical Analyses

Values are presented as means ± standard deviation (SD) from independent experiments. Intergroup comparisons were performed using the unpaired Student’s *t*-test in Excel software (ver. 2021, Microsoft, Redmond, WA, USA). One-way analysis of variance (ANOVA) was followed by the Tukey honest significant difference (HSD) test, using StatPlus Excel plug-in software (ver. 2021, StatPlus, Alexandria, VA, USA) when multiple comparisons were necessary. Differences were considered statistically significant at *p* < 0.05.

## Figures and Tables

**Table 1:** Key materials used in this study.

Reagents or Materials	Companies or Sources	Cat. Nos.	Lot. Nos.	Concentrations Used
Key antibodies				
Ant-Gap43	Santa Cruz Biotechnology (Santa Cruz, CA, USA)	sc-17790	J0920	Immunoblotting (IB), 1:10,000
Anti-Tau	Santa Cruz Biotechnology	sc-121796	J2524	IB, 1:1000
Anti-actin (also called pan-β type actin)	MBL (Tokyo, Japan)	M177-3	008	IB, 1:10,000
Anti-(pY1118)Dock7	IBL (Tokyo, Japan)	28079	002	IB, 1:250
Anti-Dock7	IBL	28057	002	IB, 1:250
Anti-hexa-histidine	MBL	D291-3	005	IB, 1:5000
Anti-rabbit IgG (goat) pre-absorbed HRP-conjugate	Nacalai Tesque (Kyoto, Japan)	21858-24	L3E2990	IB, 1:10,000
Anti-mouse IgG (goat) pre-absorbed HRP-conjugate	Nacalai Tesque	21860-61	L4B5968	IB, 1:10,000
Anti-IgG (H + L chain) (rabbit) pAb-HRP	MBL	458	354	IB, 1:10,000
Anti-IgG (H + L chain) (mouse) pAb-HRP	MBL	330	365	IB, 1:10,000
Recombinant DNAs				
pcDNA3.1(-), which is digested from pcDNA3.1(-)-shLuciferasemir	generated in this study	N/A	N/A	1.25 micrograms of DNA per 6 cm dish
pcDNA3.1(-)-shLuciferase (control) mir	Genscript (Piscataway, NJ, USA; generated in this study)	J248X396G0-2	W948408	1.25 micrograms of DNA per 6 cm dish
pcDNA3.1(-)-shDock7mir	Genscript (generated in this study)	J6826818G0-2	W947171	1.25 micrograms of DNA per 6 cm dish
pCMV5	N/A	N/A	N/A	1.25 micrograms of DNA per 6 cm dish
pCMV5-FLAG-Dock7 middle 2 domain (corresponding to amino acids 1111 to 1431 of Dock7).	[38]	N/A	N/A	1.25 micrograms of DNA per 6 cm dish
pEGFP-C1 (mamalian cell GFP expresssion plasmid)	Takara Bio (Kyoto, Japan)	not commercially available	N/A	0.625 micrograms of DNA per 6 cm dish

## Data Availability

The datasets used and/or analyzed for the current study are available from the corresponding author upon reasonable request.

## Supplementary Materials

The following supporting information can be downloaded at: https://www.mdpi.com/article/10.3390/ijms262110656/s1.
