# Supplementary material for: The ErbB2–Dock7 Signaling Axis Mediates Excessive Cell Morphogenesis Induced by Autism Spectrum Disorder- and Intellectual Disability-Associated Sema5A p.Arg676Cys
Source: Int J Mol Sci. 2025 Nov 1;26(21):10656. doi: 10.3390/ijms262110656 (PMC12608209; doi:10.3390/ijms262110656)
Supplement: Supplementary file 1 [file ijms-26-10656-s001.zip › 02.2. Supplemental figure legends.pdf]

Supplemental figure legends

Figure S1. Cells harboring Sema5A p.R676C, but not the wild type form, exhibit excessive morphological changes. (A, B) N1E-115 cells harboring either wild type Sema5A or Sema5A p.R676C were cultured for 0 or 2 days following the induction of differentiation. Representative images are shown. Cells exhibiting processes were quantitatively represented in the figure (** $p < 0.01$; n = 20 fields).

Figure S2. Expression of wild type Sema5A and Sema5A p.R676C in N1E-115 cells. Lysates of N1E-115 cells harboring either wild type Sema5A or Sema5A p.R676C, both tagged with His, were immunoblotted with an anti-His antibody.

Figure S3. Sema5A p.R676C, but not the wild type form, increases phosphorylation of Dock7. N1E-115 cells harboring either wild type Sema5A or Sema5A p.R676C were cultured for 2 days following the induction of differentiation. Transfected cell lysates were immunoblotted with the respective antibodies such as p(Y1118)Dock7 (pDock7) and Dock7.

Figure S4. Knockdown of Dock7 in cells. N1E-115 cells were transfected with plasmids encoding shDock7mir or its control (shControlmir). Transfected cell lysates were immunoblotted with the respective antibodies such as Dock7 and actin.

Figure S5. Effects of knockdown or inhibitor treatment on primary cortical neurons. (A, B) Primary cortical neurons were transfected with plasmids encoding control vector, shDock7mir, or the ErbB2 interactive domain (Middle 2 domain of Dock7) together with the plasmids encoding Sema5A p.Arg676Cys and GFP to visualize cell morphology. Cells transfected with Sema5A p.Arg676Cys and GFP were also treated with ErbB2 inhibitor I (also called inhibitor I or AG825, 0.01 mM) or Rac1 and Cdc42 inhibitor (ML-141, 0.01 mM) at 4 h post-transfection. Cells were cultured for 1 or 3 days. The morphology of the cells were represented in the diagram. Cells with processes were counted and graphically represented (** $p < 0.01$; n = 30 fields).

Figure S6. Schematic diagram of our proposed signaling pathway. The Sema5A p.R676C–induced signaling pathway is specifically mediated by ErbB2 and Dock7 to activate Rac1 and Cdc42. Whether additional putative receptors or molecules are involved in the signaling pathway from Sema5A p.R676C to ErbB2 remains to be determined in future studies. The 3D structures of mouse Sema5A, with the position of Arg-676 highlighted, and mouse Dock7 were computationally modeled and visualized using the publicly available tools AlphaFold2 and PyMOL3.

Figure S7. Computer-saved full-size gel images of blots in Figure 1.

Figure S8. Computer-saved full-size gel images of blots in Figure 2.

Figure S9. Computer-saved full-size gel images of blots in Figure 3.

Figure S10. Computer-saved full-size gel images of blots in Figure 4.

Figure S11. Computer-saved full-size gel images of blots in Figures S2, S3, and S4.
