# Supplementary figures and images for: The ErbB2–Dock7 Signaling Axis Mediates Excessive Cell Morphogenesis Induced by Autism Spectrum Disorder- and Intellectual Disability-Associated Sema5A p.Arg676Cys
Source: Int J Mol Sci. 2025 Nov 1;26(21):10656. doi: 10.3390/ijms262110656 (PMC12608209; doi:10.3390/ijms262110656)

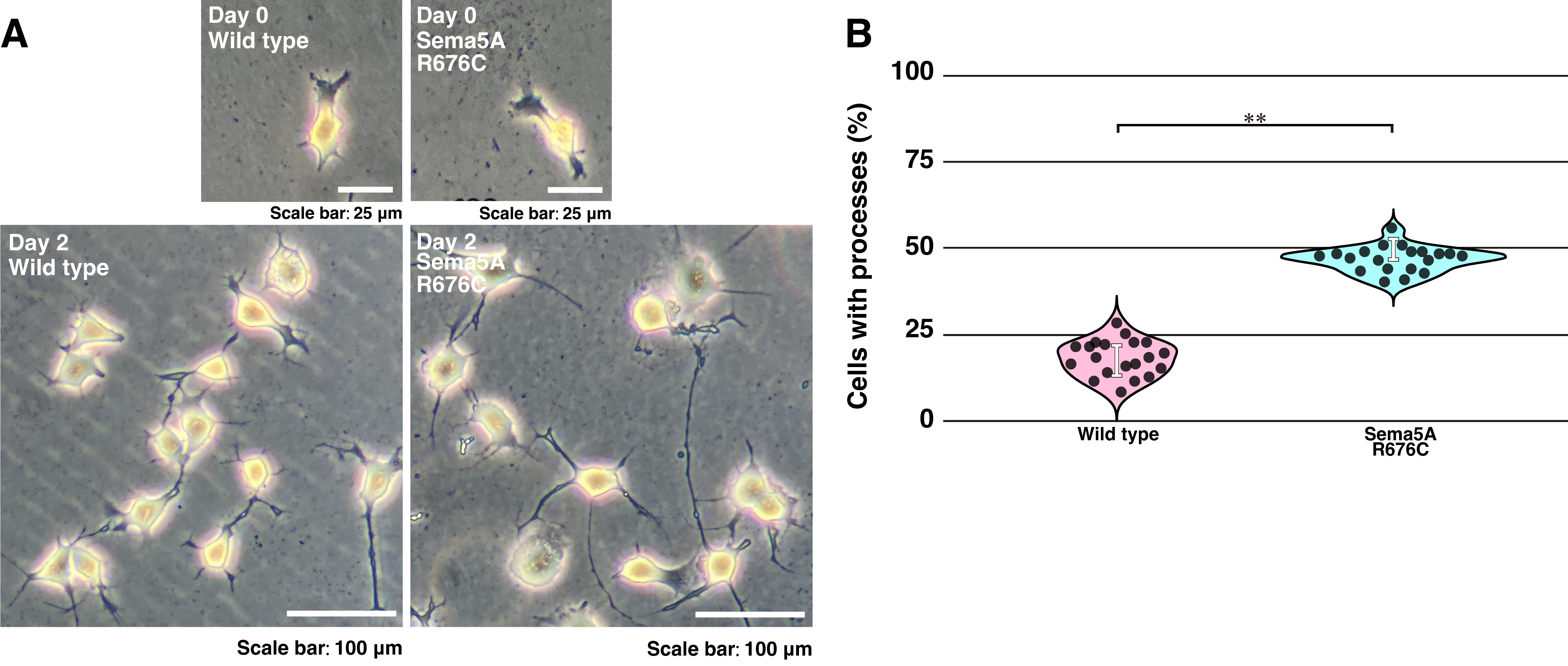

Supplement: Supplementary file 1 [file ijms-26-10656-s001.zip › Figure S1.tif]

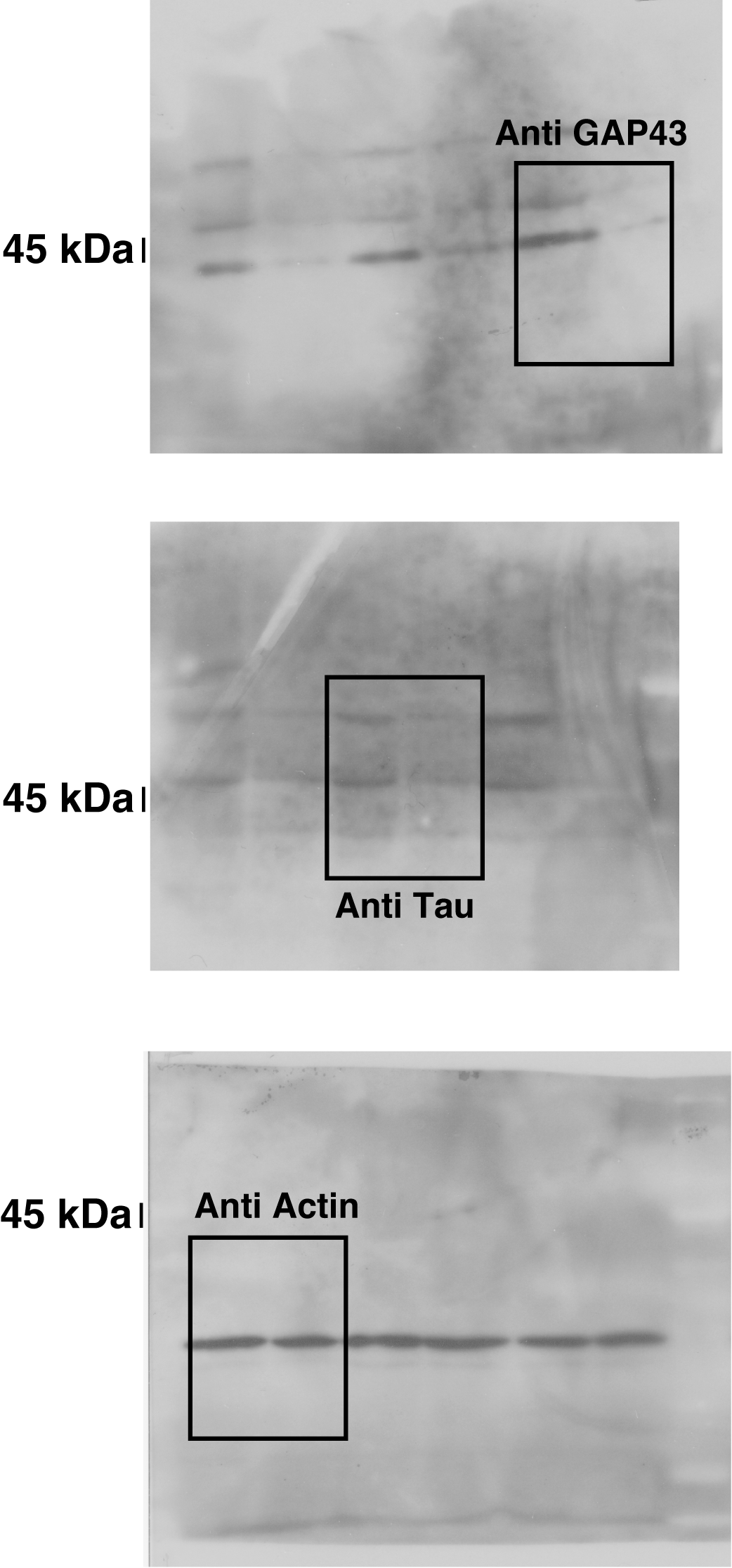

Supplement: Supplementary file 1 [file ijms-26-10656-s001.zip › Figure S10-Full gels for Figure 4.tif]

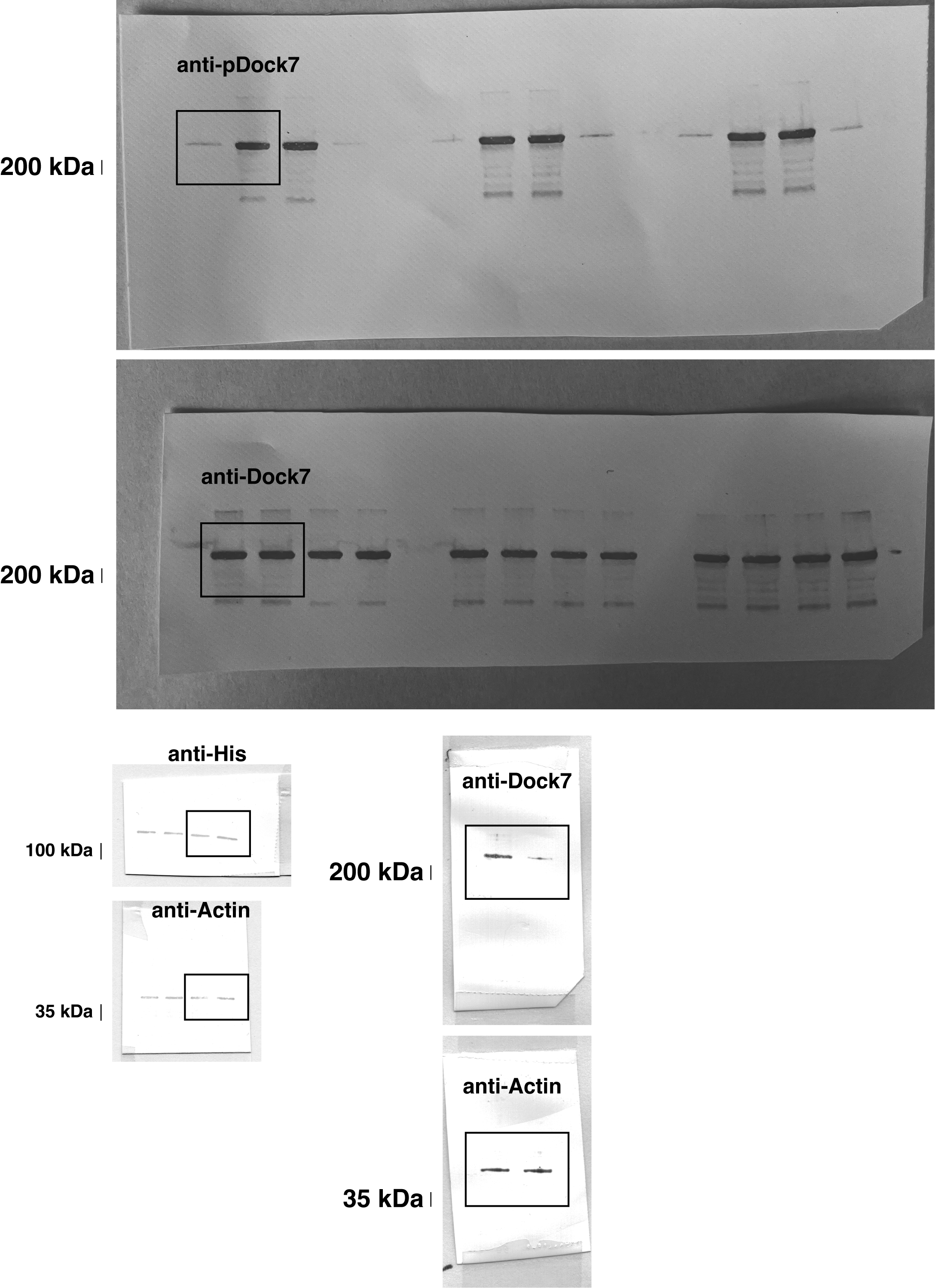

Supplement: Supplementary file 1 [file ijms-26-10656-s001.zip › Figure S11-Full gels for Figures S2 S3 and S4.tif]

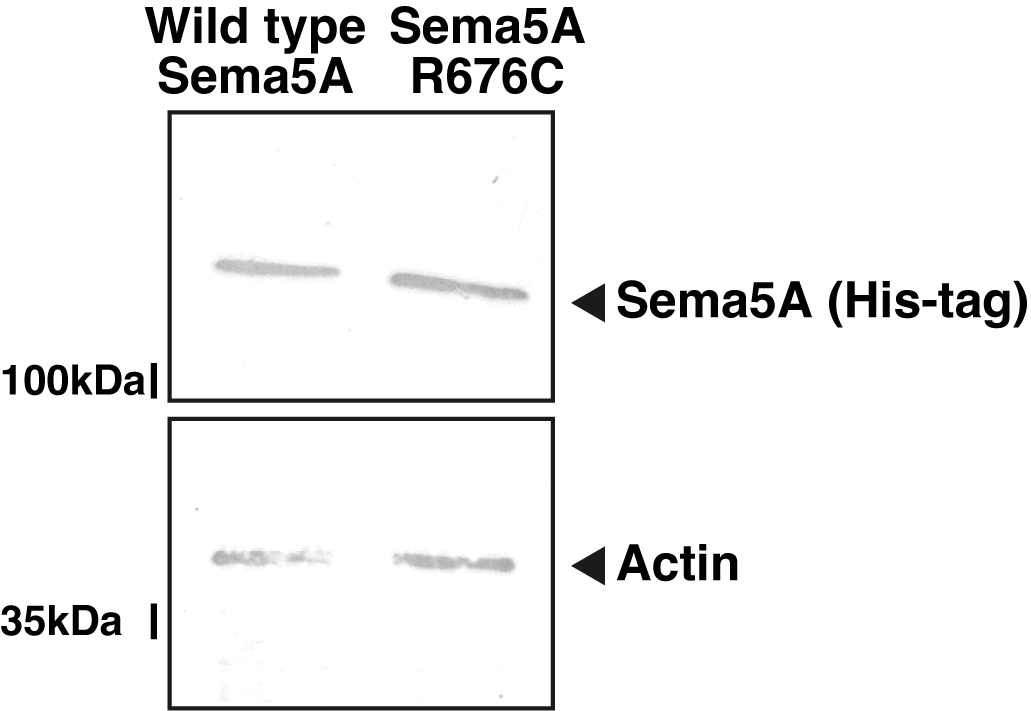

Supplement: Supplementary file 1 [file ijms-26-10656-s001.zip › Figure S2.tif]

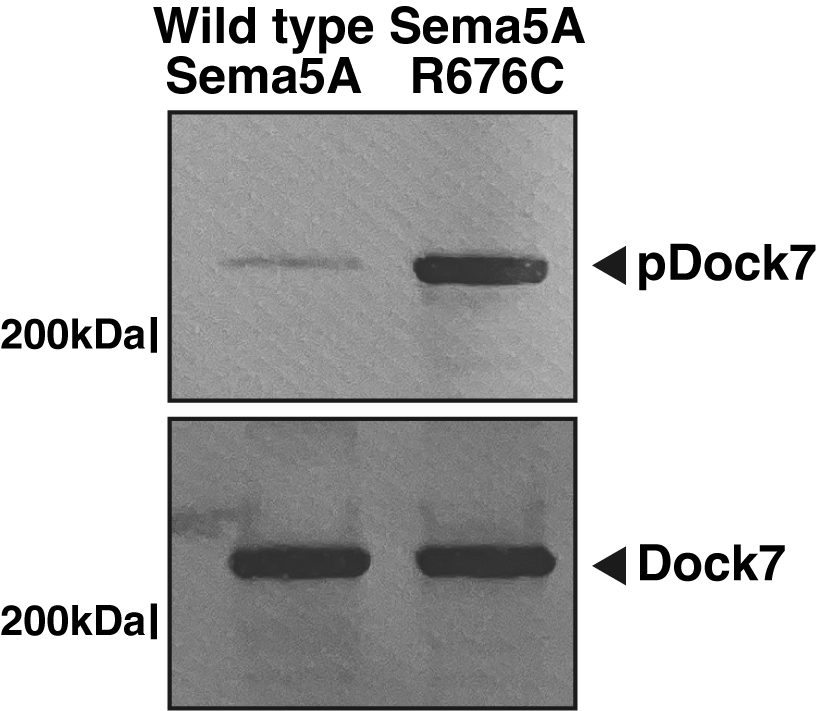

Supplement: Supplementary file 1 [file ijms-26-10656-s001.zip › Figure S3.tif]

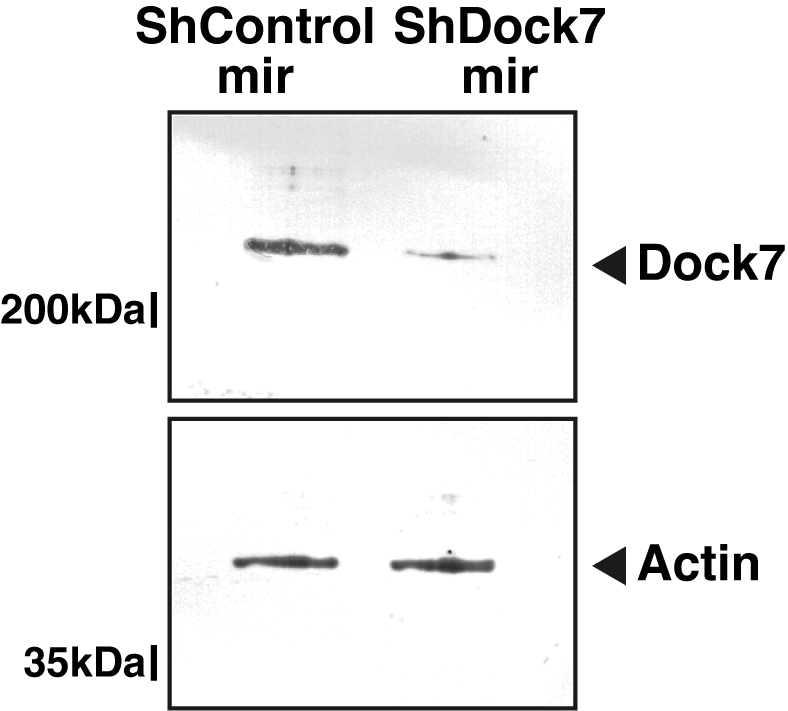

Supplement: Supplementary file 1 [file ijms-26-10656-s001.zip › Figure S4.tif]

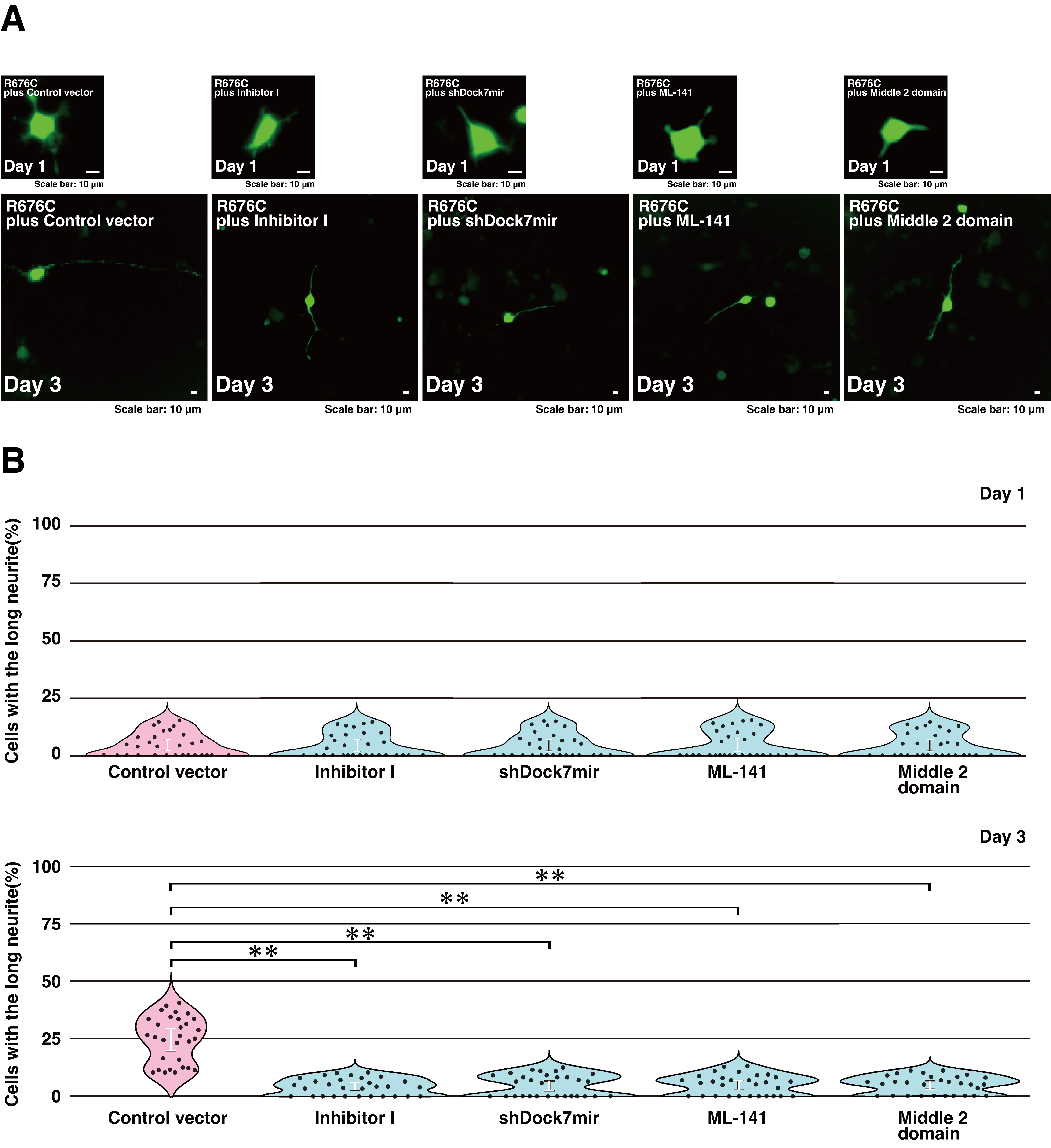

Supplement: Supplementary file 1 [file ijms-26-10656-s001.zip › Figure S5.tif]

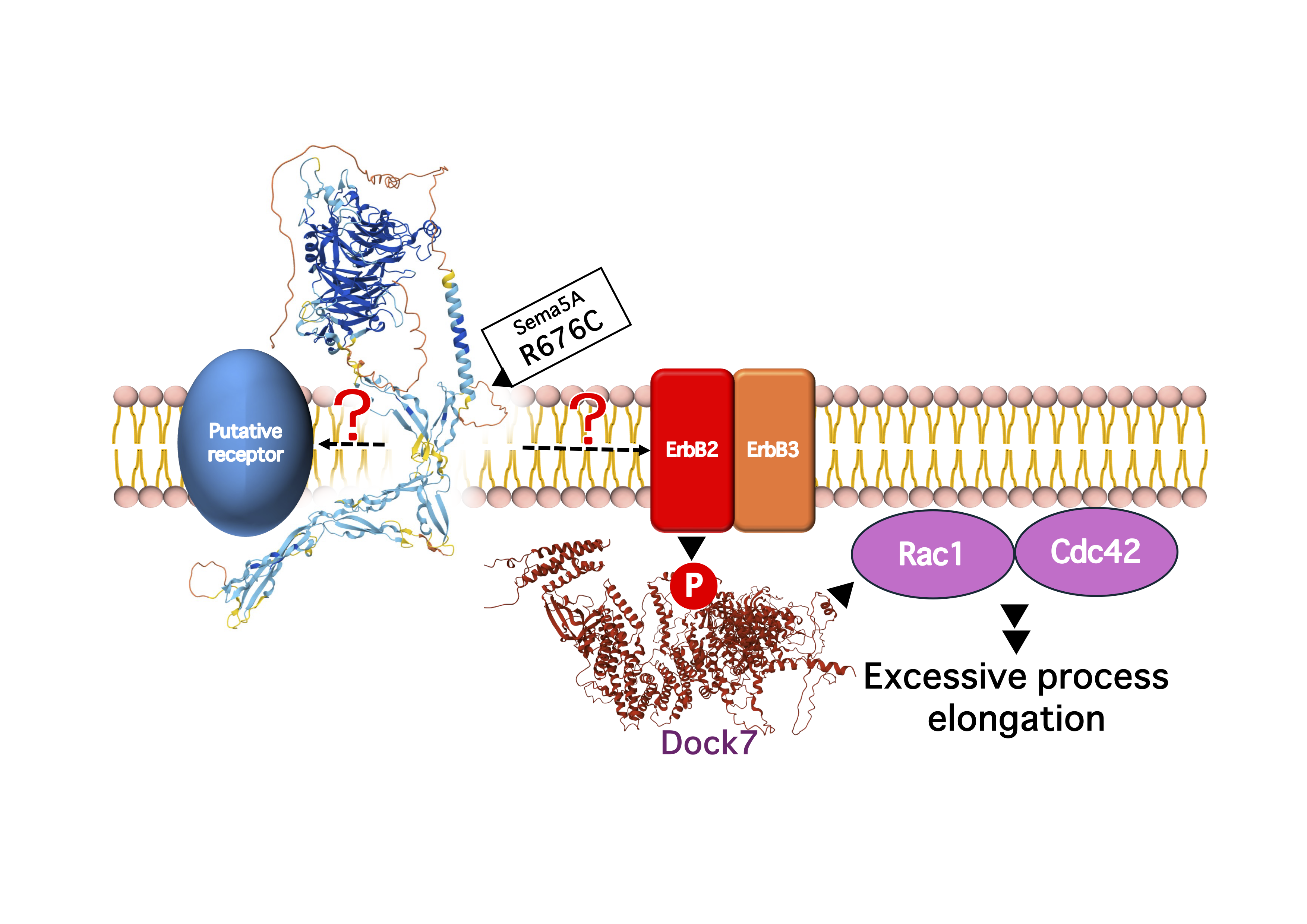

Supplement: Supplementary file 1 [file ijms-26-10656-s001.zip › Figure S6.tif]

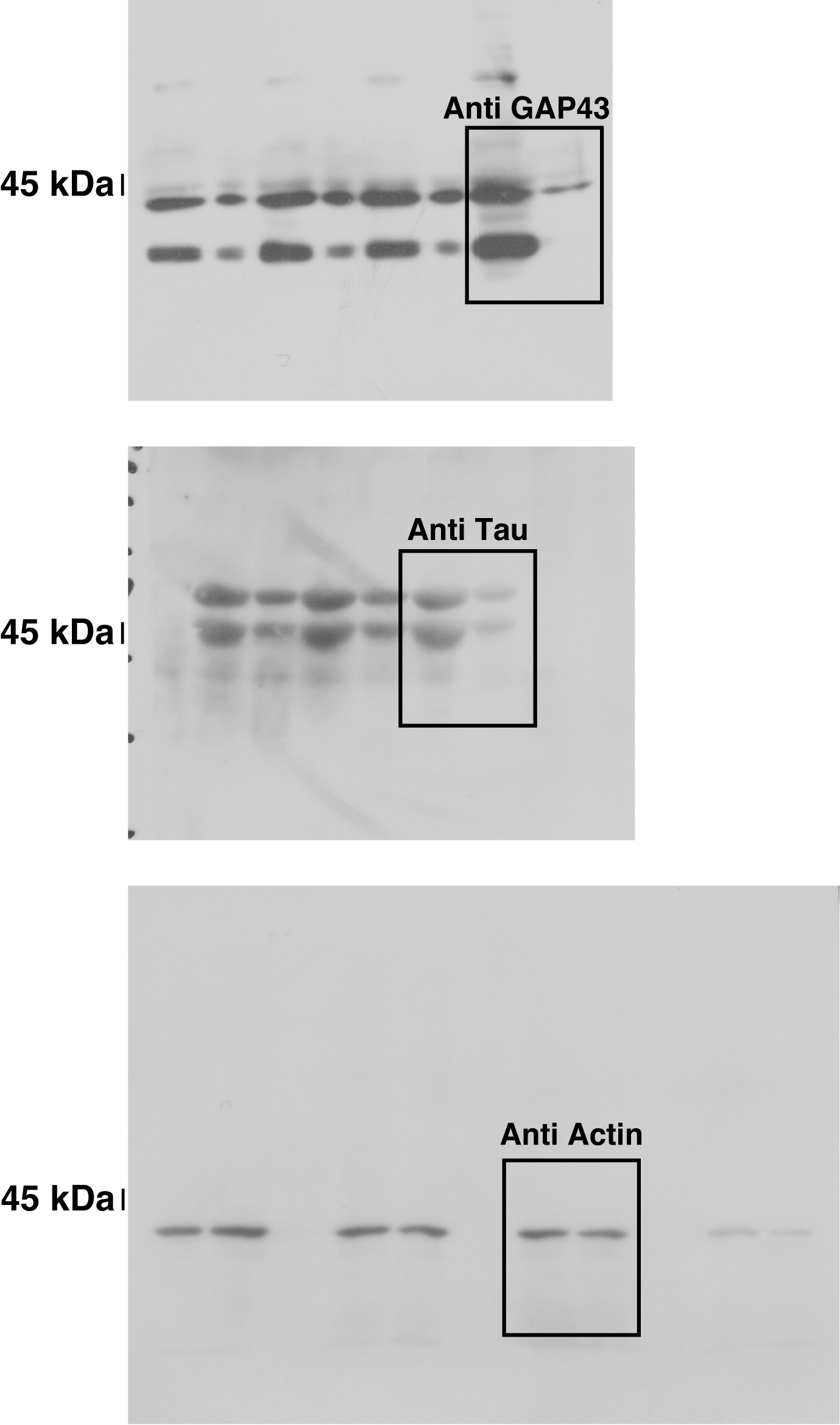

Supplement: Supplementary file 1 [file ijms-26-10656-s001.zip › Figure S7-Full gels for Figure 1.tif]

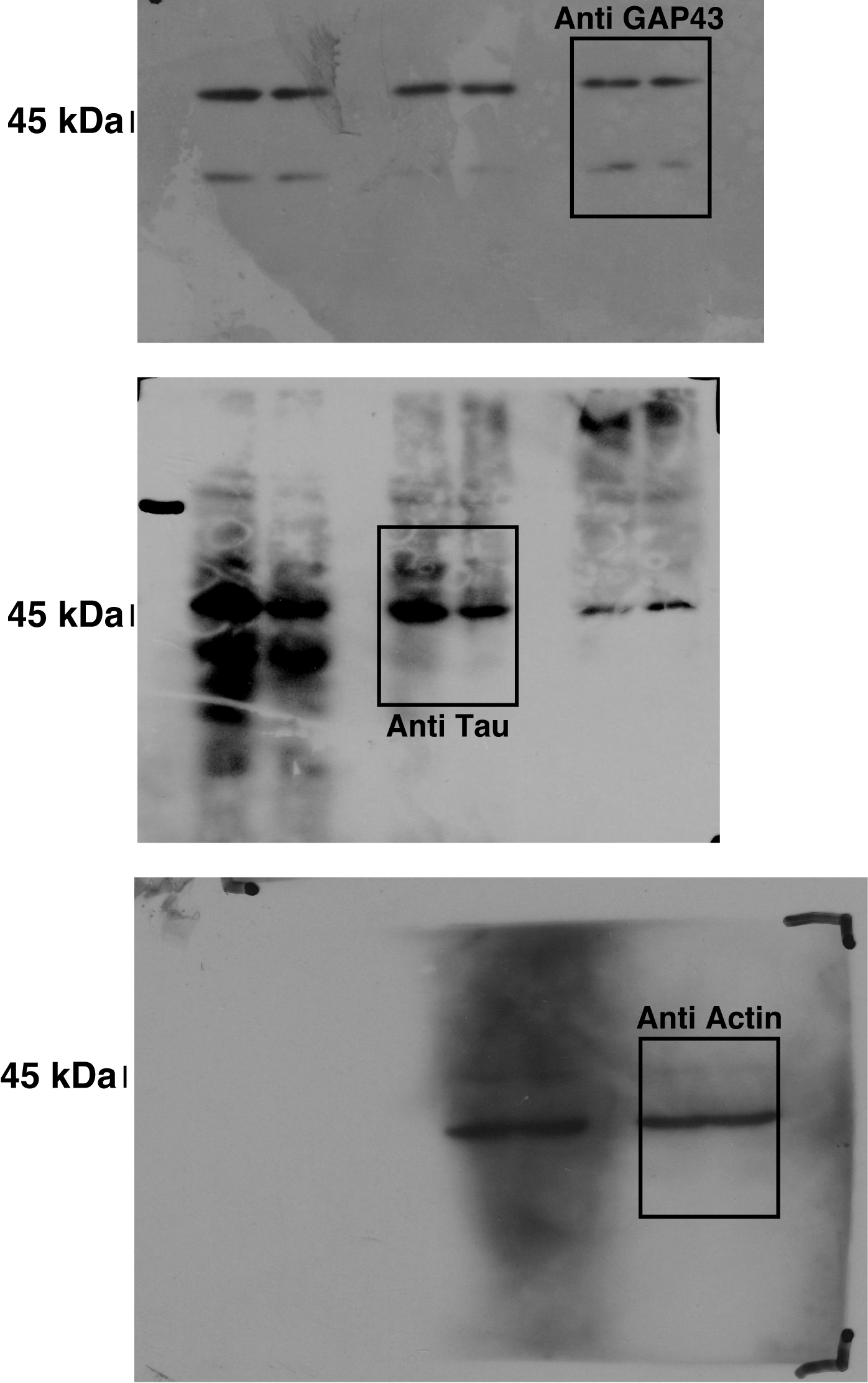

Supplement: Supplementary file 1 [file ijms-26-10656-s001.zip › Figure S8-Full gels for Figure 2.tif]

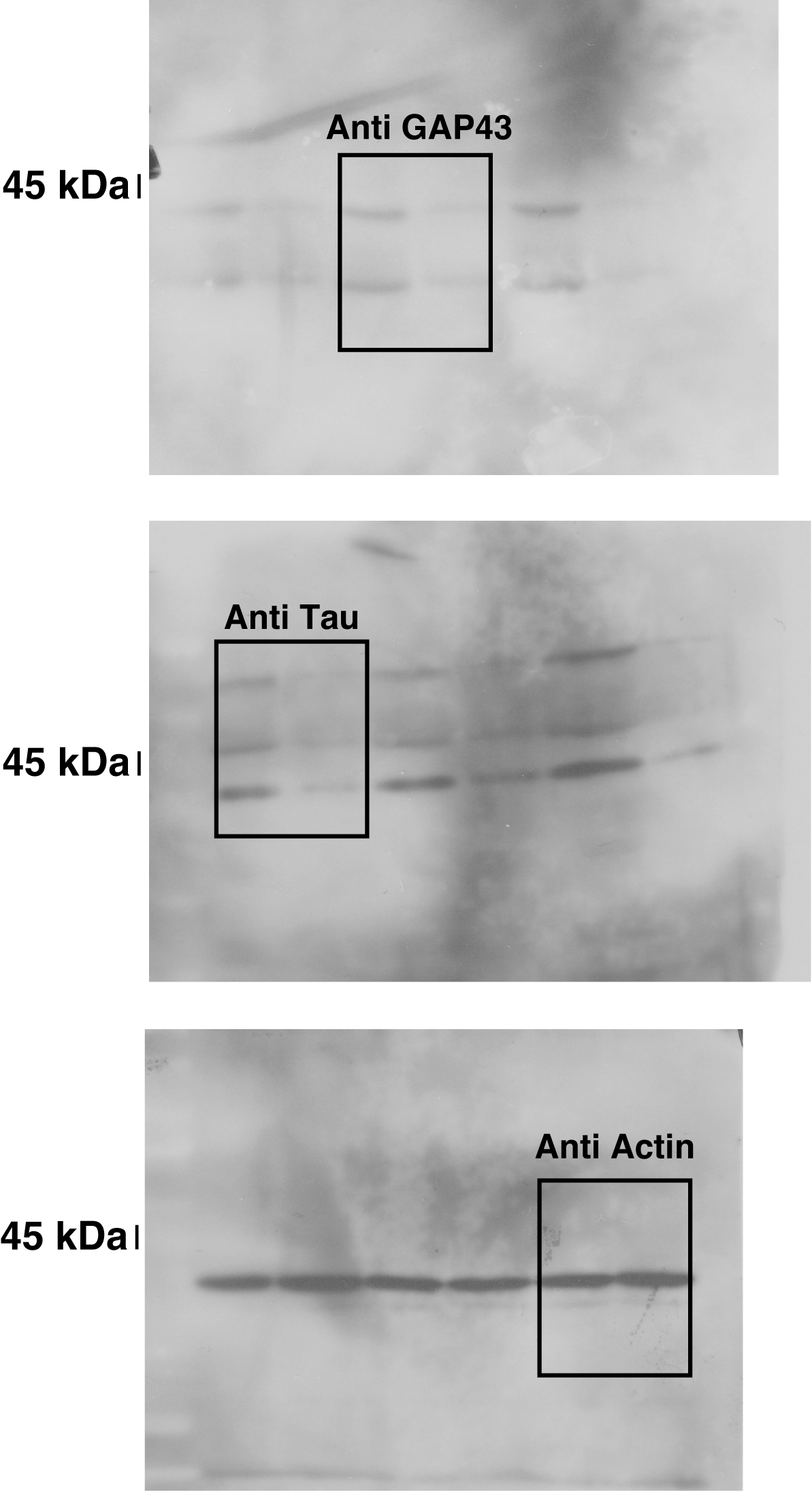

Supplement: Supplementary file 1 [file ijms-26-10656-s001.zip › Figure S9-Full gels for Figure 3.tif]
